# Selection of the *In Vitro* Culture Media Influences mRNA Expression of Hedgehog Genes, *Il-6,* and Important Genes regarding Reactive Oxygen Species in Single Murine Preimplantation Embryos

**DOI:** 10.1100/2012/479315

**Published:** 2012-07-31

**Authors:** N. Pfeifer, D. M. Baston-Büst, J. Hirchenhain, U. Friebe-Hoffmann, D. T. Rein, J. S. Krüssel, A. P. Hess

**Affiliations:** ^1^Department of OB/GYN and REI (UniKiD), Medical Center University of Düsseldorf, Moorenstr. 5, 40225 Düsseldorf, Germany; ^2^St. Elisabeth Hospital Hohenlind, Department of OB/GYN, Werthmannstr. 1, 50935 Köln, Germany

## Abstract

*Background*. The aim of this paper was to determine the influence of different *in vitro* culture media on mRNA expression of Hedgehog genes, *il-6,* and important genes regarding reactive oxygen species in single mouse embryos. *Methods*. Reverse transcription of single embryos either cultured *in vitro* from day 0.5 until 3.5 (COOK's Cleavage medium or Vitrolife's G-1 PLUS medium) or *in vivo* until day 3.5 *post coitum*. PCR was carried out for **β**-*actin* followed by nested-PCR for *shh, ihh, il-6, nox, gpx4, gpx1,* and *prdx2*. *Results*. The number of murine blastocysts cultured in COOK medium which expressed *il-6, gpx4, gpx1,* and *prdx2* mRNA differed significantly compared to the *in vivo* group. Except for *nox*, the mRNA profile of the Vitrolife media group embryos varied significantly from the *in vivo* ones regarding the number of blastocysts expressing the mRNA of *shh, ihh, il-6, gpx4, gpx1* and *prdx2*. *Conclusions*. The present study shows that different *in vitro* culture media lead to different mRNA expression profiles during early development. Even the newly developed *in vitro* culture media are not able to mimic the female reproductive tract. The question of long-term consequences for children due to assisted reproduction techniques needs to be addressed in larger studies.

## 1. Introduction

Since 1978, about 10 million children all over the world were born after applying assisted reproduction technique (ART). Therefore, 1–3% of all babies born in developed countries result from the use of ART. Besides optimizing the technique of ART to improve pregnancy rates, it is also important to consider risks of the therapy itself as well as possible long-term consequences for those children. Although human IVF procedure is constantly improved by implementation of innovative techniques and new scientific results regarding the preimplantation development, the current methods do not succeed in mimicking the *in vivo* situation completely. Therefore, knowledge about factors which regulate the preimplantation development is of great interest.

The components of the medium in which the embryo is cultured as well as the oxygen concentration of the *in vitro* culture play important roles in mimicking the female reproductive tract and therewith influence the gene expression of the preimplantation embryo [1, 2]. Development of the sequential media system already respects the changing metabolism of the preimplantation embryo with regard to glucose concentration, amino acids, and ph within its development to a blastocyst [3]. Physiologically, the preimplantation embryo develops in hypoxic condition within the reproductive tract (oxygen concentration: oviduct 8%, uterus 1.5%) whereas *in vitro* embryos are cultured with atmospheric oxygen tension (oxygen concentration: 20%). High oxygen concentration results in generation of reactive oxygen species (ROS). Besides the capability of ROS to damage cell function by modifying the structure of lipids, proteins and DNA causing strand breaks and inactivation of enzymes, ROS serve as key signalling molecules in physiological processes and are essential for embryogenesis by regulating cell proliferation and intracellular signal transduction pathways. In order to optimize the *in vitro* culture system, the oxygen concentration might be reduced by either culturing the embryo under hypoxic conditions or by adding antioxidants to the culture media.

The early embryo development from the zygote to the blastocyst stage and the implantation are complex processes with morphological and dynamic changes in the surrounding and the metabolism of the embryo. A large number of genes are involved in the early embryo development regulating immunomodulation, angiogenesis, and cell proliferation. Genes of the Hedgehog family—Sonic Hedgehog (*shh*) and Indian Hedgehog (*ihh*)—are essential for a regular early embryo development. *Shh* and *ihh* encode proteins which regulate cell proliferation and differentiation during the early embryo development and homeostasis of adult tissues [4]. *Shh acts as *a morphogen governing the pattern of neural tube development as well as patterning and midlining of somites and limbs. *Ihh* regulates ossification and is expressed in the endoderm.

The cytokine interleukin-6 (IL-6) is a well-established angiogenic factor during the implantation process of the murine embryo controlling the formation of a capillary network in the maternal decidua following embryonic implantation [5].

NADPH oxidase (NOX) producing ROS endogenously is involved in the immune defence of macrophages against bacteria. Glutathione peroxidase (GPX) and peroxiredoxin (PRDX) belonging to the cellular response against ROS are localized ubiquitously. Regulating the cell functions, PRDX plays an important role in cell differentiation, intracellular signal transduction pathways, and apoptosis [6]. For example, an upregulation of PRDX2 was demonstrated in murine testis after radiation [7].

With respect to the important roles of *shh*, *ihh,* and *il-6* during early embryo development and possible alterations in neural tube development, bone development or angiogenesis during early embryo development as well as the relevance of *nox*, *gpx4*, *gpx1,* and *prdx2* concerning ROS and feasible modifications in the antioxidant system, knowledge of their gene expression in blastocysts, and a potential influence of *in vitro* culture is of great interest. The aim of this study was to investigate *shh*, *ihh* and *il-6* and *nox*, *gpx4*, *gpx1* and *prdx2* mRNA expression in single murine blastocysts to evaluate a possible impact of *in vitro* culture compared to *in vivo* development.

## 2. Materials and Methods

### 2.1. Animals

Planning and conduction of the experimental procedures as well as maintenance of the animals was carried out in accordance to the German Guide for the Care and Use of Laboratory animals and according to the ethics board of the Heinrich-Heine University. Female, 12-week-old mice of the B6129F1 strain were obtained from Charles River Breeding Laboratories, Inc. (Wilmington, MA, USA) and maintained at 22–24°C on a 12 h light 12 h dark cycle. Female mice were superovulated by intraperitoneal (i.p.) injection of 10 IU pregnant mare serum gonadotropin (PMSG, Sigma-Aldrich, München, Germany). 48 h after PMSG, ovulation was induced by i.p. injection of 10 IU human chorionic gonadotropin (hCG, Sigma-Aldrich). Female mice were impregnated by 12-week-old fertile males of the same strain. A single male was placed with two females overnight. Mating was verified by the appearance of a vaginal plug on the following morning.

### 2.2. Zygote Recovery and Embryo Culture

For *in vitro* culturing, mated mice were sacrified by cervical dislocation 12 h after hCG injection. Ovaries and oviduct were removed and washed with universal IVF medium (Medicult, Jyllinge, Denmark) with 80 IU/mL hyaluronidase (Sigma-Aldrich). Cumulus-zygote complexes were extracted from the swollen parts of the ampulla by incubation with 80 IU/mL hyaluronidase (Sigma-Aldrich) for 3 min and gentle suction through a Pasteur pipette (Reproline Medical GmbH, Rheinbach, Germany). Cumulus-free zygotes were transferred to a culture dish (Becton Dickinson, Heidelberg, Germany) and cultured at 37°C in a humidified atmosphere of 5% CO_2_/95% ambient air. The dishes were prepared as follows: 25 *μ*L IVF medium—either COOK's Cleavage medium or the Vitrolife's G-1 PLUS medium which is supplemented with the strong antioxidant lipoate—was covered with mineral oil (Gynemed, Lensahn, Germany). Embryos, cultured in groups of 5, were observed regularly every day to monitor their development and eliminated in case of an developmental arrest. The corresponding *in vivo* blastocysts for comparison were removed 90 h after hCG injection after mice were sacrified by cervical dislocation. Uteri were flushed with a microneedle containing universal IVF medium (Medicult) under visual control. For further experiments, single blastocysts were transferred into thin wall PCR tubes (Sarstedt AG, Nümbrecht, Germany). After reverse transcription (RT), reaction single embryos were examined for mRNA-levels of **β*-actin*, *shh*, *ihh*, *il-6*, *nox*, *gpx4*, *gpx1,* and *prdx2* by two rounds of nested polymerase chain reaction (PCR) (Table 1).

### 2.3. Reverse Transcription Reaction

For each single embryo, 18 *μ*L RT mastermix was prepared containing 10 × RT buffer, 25 × dNTPs (each 100 mM), 10 × RT-random primer and DEPC-treated dH_2_O ad 18 *μ*L (High capacity cDNA Archive Kit 432217, Applied Biosystems, Foster City, CA, USA). This RT mastermix was added to a PCR tube (Sarstedt AG) with a single embryo in 1 *μ*L media, covered with light weight PCR mineral oil (Sigma-Aldrich) and kept on ice until RNA extraction. In this study complete single embryos were used. For RNA extraction, samples were heated up to 99°C for 1 min in a DNA thermocycler (UNOII, T-Gradient, Biometra GmbH, Goettingen, Germany) for total RNA release and denaturation of protein. Samples were cooled down to 4°C, and 50 U/*μ*L Moloney murine leukemia virus (MMLV) reverse transcriptase (High capacity cDNA Archive Kit 432217, Applied Biosystems) was added to each reaction. The protocol for RT was completed as follows: 25°C for 10 min, 37°C for 60 min, 4°C for ∞. After the reaction was finalized, samples were diluted with DEPC-treated dH_2_O ad 50 *μ*L and stored at −20°C until the PCR reaction was carried out.

### 2.4. Nested Polymerase Chain Reaction (PCR)

A total of 5 *μ*L for **β*-actin*, *shh*, *ihh*, *il-6*, *nox,* and *gpx1* of diluted RT product was added to 20 *μ*L of specific PCR-1-mix for **β*-actin*, *shh*, *ihh*, *il-6*, *nox,* and *gpx1*. 2 *μ*L for *gpx4* and *prdx2* of diluted RT product was added to 23 *μ*L of specific PCR-1-mix for *gpx4* and *prdx2*. The PCR Master Mix (2×) contained MgCl_2_, PCR buffer, dNTPs each 0.04 mM and 0.05 U/*μ*L *Taq*-DNA-polymerase, outer primer pair mix each 0.3 *μ*M and DEPC-treated dH_2_O ad 25 *μ*L (Fermentas, St. Leon-Rot, Deutschland). In a DNA thermocycler, the PCR-1-mix was heated to 95°C for 5 min to activate the hot-start enzyme and 40 cycles (**β*-actin*, *shh*, *ihh*, *il-6*, *nox*, *gpx4*, *gpx1* (35 cycles* prdx2*)) of 94°C for 30 sec, annealing temperature listed in Table 1 for 45 sec and 72°C for 60 sec were completed. After terminating the reaction at 72°C for 5 min, the samples were cooled down to 4°C for ∞. First round PCR products were stored at −20°C until the second PCR was carried out.

For the second PCR, 5 *μ*L (2 *μ*L) of the first round PCR product was added to 20 *μ*L (23 *μ*L) PCR-2-mix (PCR Master Mix (2×) and DEPC-treated dH_2_O ad 25 *μ*L). Program parameters were identical to the first round protocol except annealing temperatures (see Table 1). Samples were stored at −20°C until agarose gel electrophoresis was carried out. Although the use of two nested primer pairs should yield in a high specificity for the amplified cDNA, we additionally confirmed the identity of the amplicons by sequence analysis (data not shown) (biomedical research center of the Heinrich Heine University, Düsseldorf, Germany).

### 2.5. Agarose Gel Electrophoresis

In the presence of ethidiumbromide (0.5 *μ*g/mL) (Sigma-Aldrich) horizontal 2% agarose gel electrophoresis was carried out. A DNA marker (Biozym Scientific GmbH) was used to determine the sizes of the amplified fragments. The agarose gel was analyzed with the GelDoc 1000 system (Bio-Rad Laboratories, Hercules, CA, USA).

### 2.6. Statistical Analysis

Expression of the investigated mRNAs was encoded as 0, nondetectable, and 1, detectable, for each single embryo. To evaluate the statistical significance of the different mRNA expression of *in vivo* and *in vitro* cultured embryos at 90 h following hCG injection, Student's *t*-test was carried out with *P < 0.05; **P < 0.02 and ***P < 0.01.

## 3. Results

A total number of 276 single blastocysts (106 *in vivo* blastocysts, 170 *in vitro* blastocysts) were examined for **β*-actin*-, *shh*, *ihh*, *il-6*, nox, *gpx4*, *gpx1,* and *prdx2* mRNA expression. The *in vitro* blastocysts were divided into two groups; 92 blastocysts were cultured in COOK Cleavage medium and 78 blastocysts in Vitrolife G-1 PLUS. The blastocysts resulted from a total number of 140 zygotes in each medium (Table 2).

**Figure 1:**
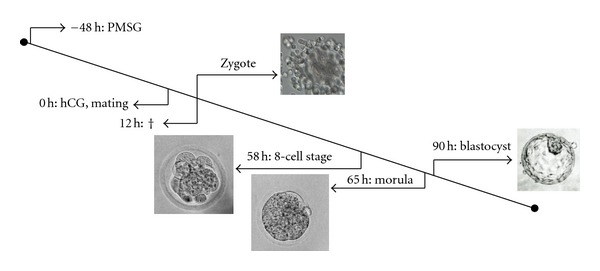
Overview representing experimental time course from stimulation of female mice, isolation of embryos, and their early development (modified after [8]).

All blastocysts—cultured *in vivo* and *in vitro*—were positive for the housekeeping gene **β*-actin* and of good quality. DEPC-H_2_O was used as negative and splenic cDNA as positive control. Only blastocysts with a regular morphology were included in the study. Every day the development of the *in vitro* cultured murine embryos was controlled and examined with an inverse microscope before the reverse transcription was performed. The experimental time course from stimulation of female mice, isolation of embryos, and their early development is shown in Figure 1.

### 3.1. mRNA Expression of *shh* and *ihh* in Single Murine Preimplantation Embryos

**Figure 2:**
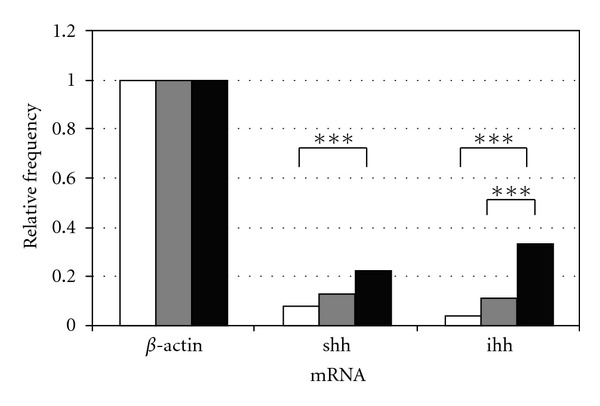
Quantitative analysis of mRNA expression of *shh,* and *ihh* of single murine blastocyst either cultured *in vivo* (white) or *in vitro* in COOK's Cleavage medium (grey) or in Vitrolife's G-1 PLUS medium (black) compared to **β*-actin* as housekeeping gene with *P < 0.05; **P < 0.02; ***P < 0.01.

All 276 single blastocysts of the three groups were examined for their mRNA expression of both *hh* genes. 8% of the *in vivo* blastocysts expressed *shh* mRNA and 4% *ihh* mRNA. In the COOK medium group 13% of *shh* mRNA and 11% of *ihh* mRNA could be detected whereas in the Vitrolife medium group 22% of the blastocysts expressed *shh* mRNA and 33% *ihh* mRNA. The *t*-test showed a highly significant difference with ***P < 0.01 for both *shh* and *ihh* in the group cultured in Vitrolife medium compared to the *in vivo* group. The blastocysts cultured in the COOK medium showed no significant difference for *shh* and *ihh* mRNA compared to the *in vivo *group. Moreover, *ihh* showed a highly significant difference of mRNA expression between both *in vitro* groups with more blastocysts expressing *ihh* mRNA in the Vitrolife group (***P < 0.01) (Tables 2 and 3, Figure 2).

### 3.2. mRNA Expression of *il-6* in Single Murine Preimplantation Embryos

**Figure 3:**
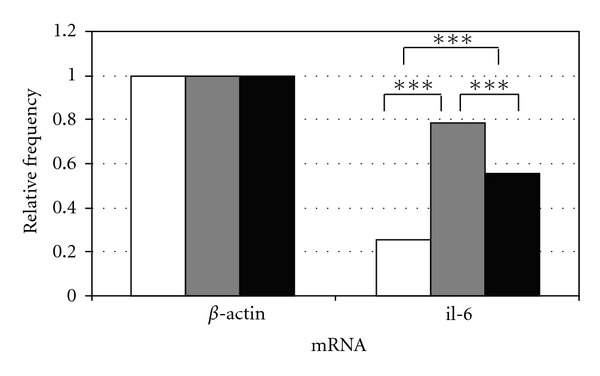
Quantitative analysis of mRNA expression of *il-6* of single murine blastocyst either cultured *in vivo* (white) or *in vitro* in COOK's Cleavage medium (grey) or in Vitrolife's G-1 PLUS medium (black) compared to **β*-actin* as housekeeping gene with *P < 0.05; **P < 0.02; ***P < 0.01.

All three collectives of single murine blastocysts were examined for *il-6* mRNA expression. 25% (*n* = 27/106) of the *in vivo* blastocysts compared to 78% (*n* = 72/92) and 55% (*n* = 43/78) of the *in vitro* groups expressed *il-6* mRNA (COOK and Vitrolife, resp.). The *t*-test showed a highly significant difference of *il-6* mRNA expression with ***P < 0.01 between both *in vitro* groups compared to the *in vivo* group as well as between the two *in vitro* groups with a higher number of blastocysts expressing *il-6* mRNA in the COOK medium group (Tables 2 and 3, Figure 3).

### 3.3. mRNA Expression of *nox*, *gpx4*, *gpx1*, and *prdx2* in Single Murine Preimplantation Embryos

The detection of *nox* mRNA transcript was comparable with no statistical significant difference in all three groups (*in vivo*: 0% (*n* = 0/106); *in vitro* COOK: 2% (*n* = 2/92); *in vitro* Vitrolife: 1% (*n* = 1/78)).

*Gpx4* mRNA transcripts could be detected in all three groups in more than 90% of the blastocysts (*in vivo*: 92% (*n* = 98/106); *in vitro* COOK: 100% (*n* = 92/92); *in vitro* Vitrolife: 99% (*n* = 77/78)], whereas the COOK medium group showed in the *t*-test a highly significant difference with ***P < 0.01 for *gpx4* mRNA compared to the *in vivo *group. In the group cultured in Vitrolife medium the *t*-test represents a high significant difference with **P < 0.02 for *gpx4* mRNA compared to the *in vivo *group.

*Gpx1* mRNA was found in 42 of 106 *in vivo* blastocysts (40%). In contrast, the *in vitro* blastocysts showed a much higher number of *gpx1* mRNA transcripts. 79 of 92 (86%) murine blastocysts of the COOK and 72 of 78 (92%) murine blastocysts of the Vitrolife medium group expressed *gpx1* mRNA.

**Figure 4:**
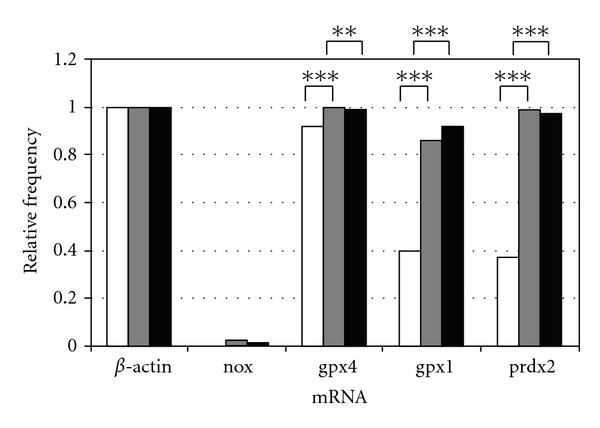
Quantitative analysis of mRNA expression of *nox*, *gpx4*, *gpx1* and *prdx2* of single murine blastocyst either cultured *in vivo* (white) or *in vitro* in COOK's Cleavage medium (grey) or in Vitrolife's G-1 PLUS medium (black) compared to **β*-actin* as housekeeping gene with *P < 0.05; **P < 0.02; ***P < 0.01.

*Prdx2* mRNA expression was comparable to the *gpx1* results with 91 of 92 (99%) blastocysts of the COOK and 76 of 78 (97%) of the Vitrolife medium group, whereas only 37% (*n* = 39/106) of the *in vivo* group blastocysts showed* Prdx* mRNA expression (all results displayed in Figure 4, Tables 2 and 3).

## 4. Discussion

A growing body of evidence in the literature suggests that *in vitro* culture of preimplantation embryos as widely used in ART is influencing the gene expression [9–14]. Moreover, different *in vitro* culture systems showed modified patterns of gene expression. Rinaudo and Schultz [1] described that important genes involved in protein synthesis, cell proliferation, and transporter functions were changed after *in vitro* culture in murine embryos compared to the *in vivo* group and the usage of two different culture media led to different gene expression profiles.

Thus, the environment of the preimplantation embryo seems to have an important impact on gene expression and embryo development.

The oxygen concentration and ROS regulate different cellular signal transduction pathways, for example, *hypoxia inducible factor* family or *nuclear factor *κ*B* and metabolic processes like glucose transport or glycolysis [15–17]. Physiologically, the early embryo development takes place in a surrounding area of 5% oxygen in comparison with an atmospheric oxygen of 20% during the *in vitro* culture in many times. Many data pointed out that the hypoxic condition of the *in vitro* culture improves the embryo development in mouse, cattle, and human compared with atmospheric tension, for example, by reducing the generation of ROS [18–21].

In order to maintain the balance of ROS and antioxidants, recently, antioxidants are added to the culture media. The addition of cysteine as a scavenger to the culture system improved the development of bovine embryos, whereas the addition of antioxidant enzymes like catalase or superoxide dismutase showed no benefit [22, 23]. As there are contradictory data concerning the addition of antioxidants to culture media, it is important to gain more insight into their relevance concerning the choice and concentration of the respective antioxidant [24–26]. 

In the present study, the majority of genes (*il-6*, *shh*, *ihh*, *gpx1*, *gpx4*,* prdx2*) showed an altered pattern of mRNA expression in the Vitrolife medium cultured blastocysts compared to the *in vivo* blastocysts. Although the *in vitro* blastocysts of the COOK medium group expressed no different *shh* and *ihh* mRNA profile compared to the *in vivo* group, the other genes showed a modified mRNA expression in the COOK medium cultured blastocysts as well. Only the *nox* mRNA expression of the *in vitro* blastocysts was similar to that of the *in vivo *group in both *in vitro *groups. These findings are concordant with the studies of Rinaudo and Schultz [1] demonstrating that not only the *in vitro* blastocysts show a modified mRNA expression pattern compared to the *in vivo* blastocysts but also that the use of varying culture media leads to a different pattern of gene expression according to the media used.

As the number of *shh* mRNA positive murine blastocysts in the COOK medium group showed no significant difference compared to the *in vivo* group, but the number of *shh* mRNA positive murine blastocysts in the Vitrolife medium group was significantly increased compared to the *in vivo* group, one can hypothesize that the components of the Vitrolife medium lead to an increased number of *shh* mRNA positive murine blastocysts. As Nguyen et al. [27] described an antiapoptotic function of SHH, the *in vitro* culture medium supplemented by antioxidants could possibly cause stress in the murine embryo resulting in an increased *shh* mRNA expression in order to avoid DNA strand breaks.

Becker et al. showed that only little *ihh* mRNA can be detected in the early murine blastocyst, which increases in concordance with the developmental process, especially with the development of the extraembryonic endoderm, where *ihh* displays a decisive function [28]. Furthermore, IHH induces the endothelial cell production in murine yolk sac tissue and plays an important role in the development of the earliest hematovascular system [29]. Although IHH exhibits important key functions considering the early development, the influence of the *in vitro* culture on *ihh* mRNA expression was not yet examined. In this study, the investigated blastocysts showed an increased number of *ihh* mRNA positive murine blastocysts in the Vitrolife medium group compared to the *in vivo* group similar to the results of the *shh* mRNA expression, whereas the number of *ihh* mRNA positive murine blastocysts in the COOK medium group showed no significant difference compared to the *in vivo* group. The significantly increased number of *ihh* mRNA positive murine blastocysts in the Vitrolife group raises concern with regard to possible disturbances of the early embryonic development. Since investigations of *ihh* *knockout* mice as well as IHH overexpression in the chicken model showed growth defects with hypertrophic chondrocytes and abnormal limbs, the significant increased number of *ihh* mRNA positive murine blastocysts in the Vitrolife medium group might influence the growth pattern of the affected blastocysts [30–32]. 

Moreover, the progesterone-dependent *ihh* production in the endometrium which increases during the first days of pregnancy marks a critical signal in uterine cell remodeling and cell proliferation during the implantation of mice and hamster [33, 34]. As *ihh* inhibits important functions as a mediator of epithelial-mesenchymal interaction in the mouse uterus during early development, a possible influence of embryonal produced *ihh*—especially with regard to the significant increased number of *ihh* mRNA positive murine blastocysts in the Vitrolife medium group—on its receptor *ptc* which is localized in the uterine mesenchyme has to be contemplated [35].

Whereas the number of *shh* and *ihh* mRNA positive blastocysts differed only in the Vitrolife medium compared to the *in vivo* group, the number of blastocysts expressing *il-6* mRNA showed a significant difference in both *in vitro* groups compared to the number of blastocysts of the *in vivo* group. Considering IL-6's function in terms of regulating angiogenic processes during early embryo development and implantation, an altered mRNA expression of *il-6* in the *in vitro* groups might lead to deviated angiogenic processes during early embryo development and implantation [5].

The four genes which are important with regard to ROS (*nox*, *gpx1*, *gpx4*, *prdx2*) were detected in both *in vitro* groups in a comparable fashion. Therefore, the supplementation of antioxidants seemed to have no beneficial effect at least on the mRNA expression of the representative genes investigated. Since Gonçalves et al. [23] described that addition of antioxidants to IVF media impairs early development of bovine embryos, implications of the murine embryo development due to the antioxidant enriched media have to be considered especially if an anticipated effect on ROS depending genes is not evident. The increased number of mRNA positive murine blastocysts in both *in vitro* collectives regarding *gpx4*, *gpx1,* and *prdx2* might be explained with an adaptive mechanism of the embryo because of possible stress in the modified culture conditions compared with the physiological conditions in the female reproductive tract. Certainly it would have been interesting to investigate these expression patterns in a culture system with reduced oxygen concentration but could not do it due to technical reasons. 

In summary, the present study shows that the *in vitro* culture alters the mRNA expression pattern of single murine embryos during early development. In spite of the constant effort to optimize the *in vitro* culture media, up to today there is still no perfect imitation of the physiological conditions within the female reproductive tract. Moreover, different *in vitro* culture media lead to a different gene expression profile in single murine blastocysts which might result in a disturbed early mouse development. Therefore, possible implications of the *in vitro* culture regarding modified pattern of mRNA expression should raise concern in terms of long-term clinical aspects and health condition of children born after ART and hence need to be addressed in further studies. In parallel more effort in order to optimize the *in vitro* culture media is needed.

## Figures and Tables

**Table 1:** Sequences of oligonucleotides and annealing temperature used in reverse transcription and nested-PCR.

mRNA	*Nested *PCR	*Annealing *temperature (^°^C)	Size of the fragment (bp)	3^′^-/5^′^-end	Sequence of oligonucleotide 5^′^→3^′^
**β*-actin *X03672		54	407	5^′^	CAA GGT GTG ATG GTG GGA ATG G
				3^′^	CAG GAT GGC GTG AGG GAG AGC A
*shh* NM_009170	Outer pair	55	530	5^′^	GAG ACC CAA CTC CGA TGT GT
				3^′^	GAT GTC GGG GTT GTA ATT GG
	Inner pair	56	150	5^′^	CTG GCC AGA TGT TTT CTG GT
				3^′^	CTC GGC TAC GTT GGG AAT AA
*ihh* NM_010544	Outer pair	58	438	5^′^	CAC TTG TGG TGG AGA TGT G
				3^′^	TAC CAC ACG CTT GTC AGC TC
	Inner pair	53	106	5^′^	CTC TAA CCA CTG CCC TCC TG
				3^′^	AGA GGA CGG AGA CAA CCT CA
*il-6* NM_031168	Outer pair	56	339	5^′^	GTT CTC TGG GAA ATC GTG GA
				3^′^	GGA AAT TGG GGT AGG AAG GA
	Inner pair	56	226	5^′^	TGT GCA ATG GCA ATT CTG AT
				3^′^	CTC TGA AGG ACT CTG GCT TTG
*nox* NM_172203	Outer pair	59	683	5^′^	GGG ATG ACC ATA AGG GGA GT
				3^′^	CCA GCC AGT GAG GAA GAG AC
	Inner pair	59	216	5^′^	TCC ATT TCC TTC CTG GAG TG
				3^′^	CCC AAC CAG TAC AGC CAC TT
*gpx4* NM_001037741	Outer pair	58	410	5^′^	AGT ACA GGG GTT TCG TGT GC
				3^′^	CGG CAG GTC CTT CTC TAT CA
	Inner pair	57	166	5^′^	CCG GCT ACA ACG TCA AGT TT
				3^′^	ACG CAG CCG TTC TTA TCA AT
*gpx1* NM_008160	Outer pair	58	847	5^′^	GTC CAC CGT GTA TGC CTT CT
				3^′^	TCG ATG TCG ATG GTA CGA AA
	Inner pair	45	278	5^′^	GGT TCG AGC CCA ATT TTA CA
				3^′^	GGG CAC GCG TCC ATG TCG GC
*prdx2* NM_011563	Outer pair	56	946	5^′^	TAG GGC TCT CTC GGT TTT GA
				3^′^	TTG ACT GTG ATC TGG CGA AG
	Inner pair	56	179	5^′^	GGT GCC TTC AAG GAA ATC AA
				3^′^	GCC TAG CTT TCG GAA GTC CT

**Table 2:** Number of zygotes developed to blastocyst in correlation with gene expression of the mRNAs of interest.

	*In vivo*	*In vitro* in COOK cleavage medium	*In vitro* in Vitrolife's G-1 PLUS medium
Number of zygotes	unknown	140	140
Number of good quality blastocysts	106	92	78

No. of blastocyst with detectable mRNA expression of			
**β*-actin*	106/106	92/92	78/78
* shh*	8/106	12/92	17/78
* ihh*	4/106	10/92	26/78
* il-6*	27/106	72/92	43/78
* nox*	0/106	2/92	1/78
* gpx4*	98/106	92/92	77/78
* gpx1*	42/106	79/92	72/78
* prdx2*	39/106	91/92	76/78

**Table 3:** Frequency of detection of **β*-actin, shh, ihh, il-6, nox, gpx4, gpx1,* and *prdx2* mRNA in single murine blastocyst with **β*-actin* as internal standard.

Specific mRNA expression (%)	**β*-actin *	*shh*	*ihh*	*il-6*	*nox*	*gpx4*	*gpx1*	*prdx2*
*In vivo* cultured embryos	100	8	4	25	0	92	40	37
*In vitro* cultured embryos (COOK)	100	13	11	78	2	100	86	99
*In vitro* cultured embryos (Vitrolife)	100	22	33	55	1	99	92	97
